# Effect of Spatial and Temporal Urban Isolation on the Genetic Diversity, Acoustic Variation, and Morphological Characteristics of an Urban Survivor Bird Species

**DOI:** 10.1002/ece3.70972

**Published:** 2025-04-28

**Authors:** Luis Cueva, Eric J. Fuchs, Gilbert Barrantes, Ruth Madrigal‐Brenes, Luis Sandoval

**Affiliations:** ^1^ Escuela de Biología Universidad de Costa Rica San José Costa Rica; ^2^ Programa de Posgrado en Biología, Sistema de Estudios de Posgrado Universidad de Costa Rica San José Costa Rica; ^3^ Universidad Estatal Amazónica Puyo Ecuador; ^4^ Centro de Investigación en Biodiversidad y Ecología Tropical (CIBET) Universidad de Costa Rica San José Costa Rica; ^5^ Museo de Zoología, Centro de Investigación en Biodiversidad y Ecología Tropical (CIBET) Universidad de Costa Rica San José Costa Rica

**Keywords:** bioacoustic, Costa Rica, genetic structure, *Melozone leucotis*, SSR

## Abstract

Urbanization modifies ecosystems by fragmenting natural habitats and increasing isolation between populations. Therefore, a reduction in gene flow among isolated populations is expected with greater distance and time since fragmentation. Changes in the structure, density, or community composition of the remaining habitats often result in species' differences in acoustic and morphological traits. However, the relationship between genetics, vocalizations, and morphological divergence in urban areas over time remains poorly understood. We analyzed ten years of genetic, acoustic, and morphological data from isolated populations of the white‐eared ground‐sparrow. We recorded and measured five acoustic traits, six morphological traits, and used seven microsatellites (SSRs) to compare the effect of urban expansion on the acoustics, morphology, and gene flow patterns across populations over a 10‐year period. We found an increase in inbreeding, song duration, number of elements, and frequency of maximum amplitude, but a decrease in female body size and changes in male beak, decreasing size in one population and increasing in another. In general, we found changes in all characteristics studied but only found a significant correlation between genetic diversity and the acoustic characteristics of songs. Our results corroborate that urbanization acts as an important barrier for white‐eared ground sparrows, which leads to significant divergence in genetic and behavioral traits.

## Graphical abstract

Urbanization fragments habitats and isolates populations, reducing gene flow and driving divergence in genetic, acoustic, and morphological traits. Over ten years, isolated populations of white‐eared ground‐sparrows exhibited increased inbreeding, altered song characteristics, and changes in body and beak size, with significant correlations found between genetic diversity and acoustic traits. These findings highlight urbanization as a critical barrier shaping genetic and behavioral divergence in this species.

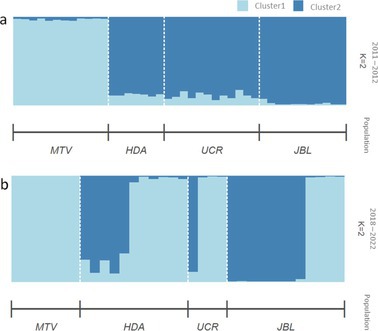

## 1 Introduction

Urban development results in rapid environmental changes (Grimm et al. 2008; Johnson and Munshi‐South 2017) that transform natural habitats into small patches of vegetation surrounded by a matrix of man‐made constructions such as buildings and roads. These fragments with depauperate resources are also frequently highly contaminated by noise, lights, or solid waste (Fahrig and Rytwinski 2009; Biamonte et al. 2011). The reduction in natural habitats isolates bird populations, as urban development restricts or prevents the movement of individuals across populations (Lynch and Baker 1994; Crooks et al. 2004). It also hinders gene flow, which, consequently, may reduce the genetic diversity of populations (Lynch and Baker 1994; Johnson and Munshi‐South 2017). In birds, changes in behavior (e.g., vocalizations, nest construction, predator responses) and morphology (e.g., bill and body size) have also frequently been reported as a consequence of different selective pressures acting on isolated populations (e.g., sexual selection, predation, environment) (Brumm 2004; Foster et al. 2008; Luther and Derryberry 2012; Sandoval et al. 2014; Geffroy et al. 2020; Corrales‐Moya et al. 2021; Méndez et al. 2020).

Increased isolation between populations and the effect of genetic drift and inbreeding on small populations are the key drivers of genetic diversity loss within populations and an increase in spatial genetic structure in urban isolated bird populations (Keller and Waller 2002; Whitlock 2004; Johnson and Munshi‐South 2017; Miles et al. 2019). In wren‐tits, *Chamaea fasciata* , significant genetic structure was found in populations separated by a highway and a strip of residential development in southern California (Delaney et al. 2010). In the song sparrow *Melospiza melodia* , researchers found lower genetic diversity in populations where urban development had been extensive and had occurred over long periods of time (Unfried et al. 2013). These results indicate that urbanization has the potential to decrease genetic diversity within fragmented populations inside urbanized matrices and increase the genetic structure of bird populations.

The genetic structure detected in urban populations of several bird species correlated with song differences (González et al. 2011; Purushotham and Robin 2016; Graham et al. 2017). Variation in the acoustic traits of songs among populations emerges in response to different habitat characteristics (e.g., vegetation density or noise levels). These changes presumably improve sound transmission and communication across noise‐polluted environments (Morton 1975; Boncoraglio and Saino 2007; Ey and Fischer 2009; Hardt and Benedict 2021). Acoustic variations may also result from acoustic drift. This occurs when birds randomly develop distinct repertoires or exhibit random differences in songs that are learned and subsequently reproduced (Baker and Cunningham 1985; Wilkins et al. 2013), as it has been observed in populations of Darwin's finches (Grant and Grant 2002). In this process, young birds introduce “learned errors” into the adult acoustic repertoire, and repertoires are further shaped by the random extinction of song types (Grant and Grant 2002). These changes in songs within populations can persist or even amplify over time, as songs are learned and transmitted across generations (Warren et al. 2006). Hence, the correlation between genetic diversity and the diversity of song dialects may arise when the survival and reproductive success of individuals with some particular genotypes learn and produce the same vocal repertoire (Danielson‐François et al. 2006; Irwin et al. 2008).

Variations in song acoustic characteristics (e.g., frequency and duration) have also been related to individuals' body and beak sizes (Podos 2001, 2010). Larger birds with larger beaks tend to produce lower frequency songs (Wallschläger 1980; Bradbury and Vehrencamp 1998; Fletcher and Tarnopolsky 1999; Slabbekoorn and Smith 2002a; Brumm 2009). To produce low‐frequency songs, birds require a larger medial syringeal labia and an enlarged upper portion of the esophagus, and both are constrained by body size (Riede et al. 2006; Riede and Goller 2010). For example, in purple‐crowned fairy‐wrens ( *Malurus coronatus* ), barn swallows ( *Hirundo rustica* ), and black swans ( *Cygnus atratus* ), individuals with larger body sizes produced lower song frequencies (Galeotti et al. 1997; Patel et al. 2010; Hall et al. 2013). In house finches, *Haemorhous mexicanus* from Arizona, urban individuals have longer and deeper beaks than rural birds and emit songs with slower trills and a wider frequency range (Badyaev et al. 2008).

White‐eared ground‐sparrows, *Melozone leucotis* , are urban‐avoiding birds (Rodríguez‐Bardía et al. 2022) that inhabit the central valley of Costa Rica in isolated forested settlements embedded inside the great urban area (Sandoval et al. 2015; Juárez et al. 2020; Rodríguez‐Bardía et al. 2022). Previous work on this species reported that urbanization limits the movement of individuals and gene flow between populations due to barriers and isolation by distance, resulting in significant genetic structuring among Costa Rican populations (Rodríguez‐Bardía et al. 2022). Additional evidence of the effect of barriers and distance is provided by the banding program of this species since. Nearly all birds of different populations have been banded since 2011; however, not a single individual of a population has been recorded in other populations. Previous research has also identified changes in song dialects among different populations (Sandoval et al. 2014, 2016; Bonilla‐Badilla 2021). Additionally, differences in song frequencies and duration across populations have been attributed to anthropogenic noise levels. Specifically, males in populations exposed to higher levels of noise tend to exhibit an increase in both the minimum (Juárez et al. 2022) frequency and duration of their songs (Sandoval et al. 2014, 2015, 2016; Juárez et al. 2020; Méndez et al. 2020; Rodríguez‐Bardía et al. 2022). These studies suggest that genetic and cultural traits are under intense selective pressures imposed by urbanized environments. However, it is yet unknown how these traits change over time and how they track population dynamics.

In Costa Rica, urban development began before the 1900s but expanded rapidly after the 1970s, resulting in the loss and fragmentation of significant portions of natural environments (Biamonte et al. 2011). In the Costa Rican Central Valley, the most urbanized area in the country, natural environments were replaced by shade coffee plantations, an agricultural system still suitable for a large number of birds and other taxa. However, coffee plantations and secondary growth areas were reduced by 41%, and urban landscape increased by 37% between 1973 and 2006 (Biamonte et al. 2011). The urban expansion continued, and by 2024, 83.3% of Costa Rican human population concentrated in cities or surrounding metropolitan areas (Joyce 2006; World Population Review 2024). Therefore, over the past decades, urban expansion in Costa Rica has significantly reduced natural and semi‐natural habitats, leading to increased isolation of bird populations (Joyce 2006; Biamonte et al. 2011), the effects of which are not yet known. Considering the rapid expansion of the urbanscape, we propose as the main objective of this study to describe changes in genetic diversity, vocalization, and morphology and their relationship over a 10‐year period in populations of white‐eared ground‐sparrow isolated by urban development in Costa Rica. We hypothesize that differences in genetic diversity, vocalizations, and morphology will correlate with the time populations have been separated, as population divergence increases with isolation imposed by urban barriers. We predict lower heterozygosity, higher inbreeding, and genetic structure in the same populations after 10 years of isolation. We also predict a reduction of minimum frequency in songs due to an increase of urban noise (Méndez et al. 2020) after 10 years of accelerated urban expansion. Finally, we predict a reduction in some morphological features (e.g., bill dimensions, wing length) due to a reduction of food sources and an increase in competition with urban exploiter species (Huyser et al. 2000; Mennechez and Clergeau 2006) as urbanization increases over the years.

## 2 Material and Methods

**FIGURE 1:**
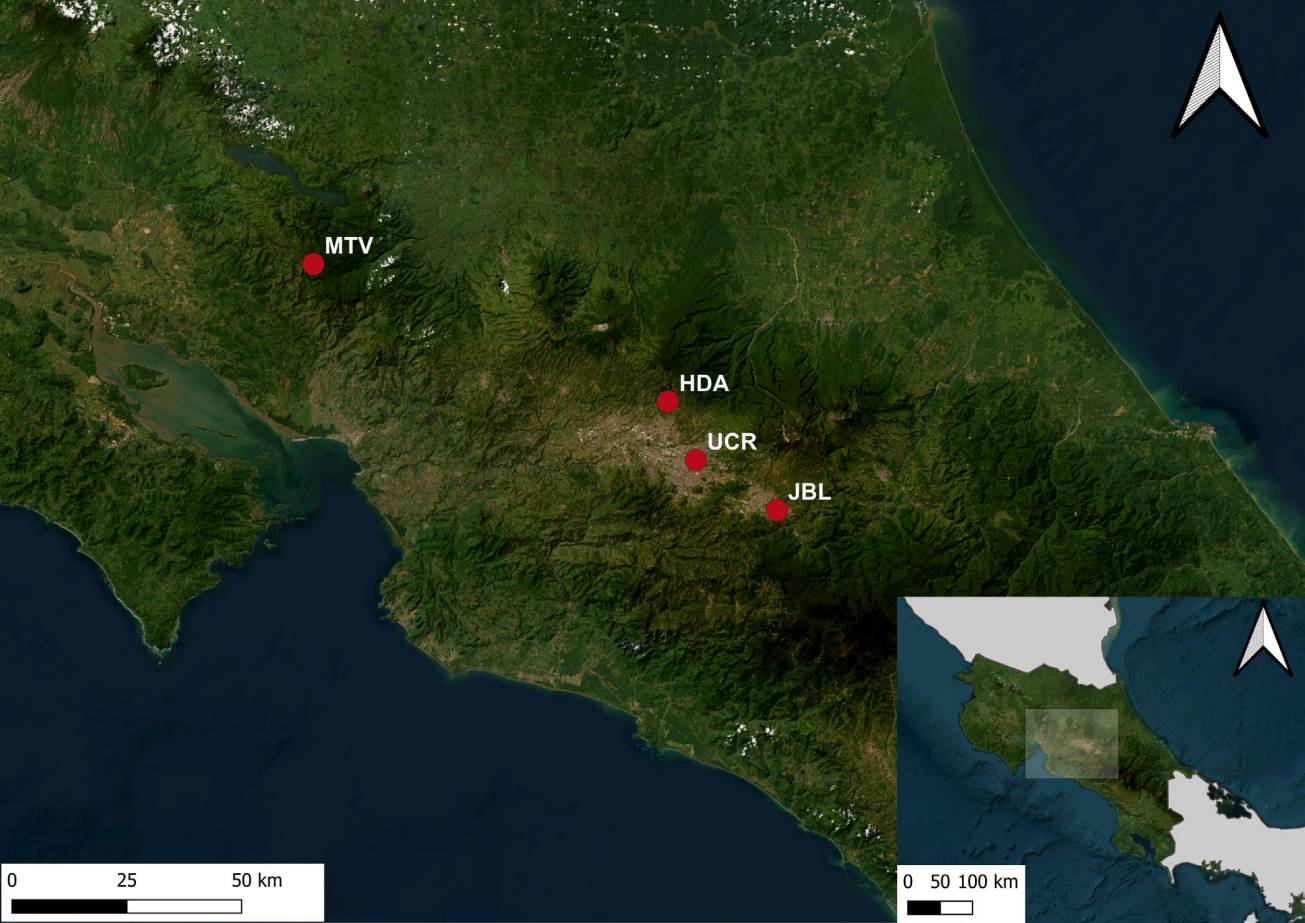
Satellite image showing the four sampled locations for white‐eared ground‐sparrow (red dots) in Costa Rica. HDA, Getsemaní; JBL, Jardín Botánico Lankester; MTV, Estación Biológica Monteverde; UCR, Universidad de Costa Rica.

We conducted this study in four populations of white‐eared ground‐sparrows in Costa Rica (Figure 1): (1) Estación Biológica Monteverde (MTV), Puntarenas province (10°18′ N, 84°48′ W, 1600 m), characterized by coffee plantations and large forest patches that connect with more extensive mature forests (Rodríguez‐Bardía et al. 2022). (2) Getsemani (HDA), Heredia province (10°01′ N, 84°05′ W, 1350 m); it is a non‐urbanized site, with secondary forest patches and abandoned coffee plantations and dense thickets with little human presence (Juárez et al. 2020). Urbanization started to expand from the edges of the study area since 2000. (3) Jardín Botánico Lankester (JBL), Cartago province (9°50′ N, 83°53′ W, 1370 m); it includes a secondary forest, gardens, and buildings, with little disturbance and human presence, but immersed in an urban matrix that has rapidly extended since 1990 (Juárez et al. 2020). (4) The Universidad de Costa Rica (UCR), San José province (09°56′ N, 84°05′ W, 1200 m), is a highly urbanized site, with a secondary forest reserve surrounded by buildings, open areas, and gardens, and it is exposed to intense human disturbance (Juárez et al. 2020).

### 2.1 Historical and Current Sampling

Historical acoustic and morphological data were collected in 2011 and 2012 and were deposited in the Laboratorio de Ecología Urbana y Comunicación Animal (LEUCA), Universidad de Costa Rica. These data are part of a long‐term research focus on analyzing the effect of urbanization on the behavior, morphology, and genetic diversity of bird populations. To obtain historic genetic diversity estimates, we used blood samples collected in 2011–2012. We will refer to the historical 2011–2012‐year sampling as “period 1”.

Current acoustic and morphological data were collected from 2018 to 2022. We recorded each banded male using a Marantz PMD661 digital recorder and a Sennheiser ME66/K6 unidirectional microphone. The recordings were stored in WAV format with a sample rate of 44.1 kHz and a precision of 24 bits. All recordings were conducted between 4:55 and 6:00 h when this species is most vocally active (Sandoval et al. 2016). We captured individuals using mist nets (12 × 2.5 m, 15 mm mesh eye) inside each pair territory. We provided each captured individual with a unique numbered metal ring and a color combination of plastic rings. From each bird, we obtained 10 μL of blood from the brachial vein and stored it in 95% ethanol or lysis buffer for molecular analyses (Seutin et al. 1991; Rodríguez‐Bardía et al. 2022). We will refer to the period 2018–2022‐year sampling as “period 2”.

All procedures were carried out in accordance with Costa Rican legislation. Research permits and animal care protocols were granted to LS, the principal investigator, by the Animal Care Committee (ACC) of the University of Costa Rica and the Research Committee of the Biology School.

### 2.2 DNA Extraction and SSR Amplification

We extracted DNA from blood samples using the DNeasy blood and tissue kit (Qiagen Inc., Valencia, CA, USA) following the manufacturer's protocol. Microsatellites (SSRs) were chosen as markers for genetic diversity analysis. We used seven primers: Mme2, Mme7, Mme8, Asμ15, Escμ6, Gf01, and Gf05 (Petren 1998; Jeffery et al. 2001; Bulgin et al. 2003), which have been previously shown to be polymorphic and amplified well for this species (Rodríguez‐Bardía et al. 2022). We followed the Qiagen Multiplex master kit (Qiagen Inc., Valencia, CA) procedure to amplify the SSRs. All markers were amplified through Polymerase Chain Reactions (PCR) using a 10 μL reaction, containing 2 μL of 0.4 μM primer mix, 1.5 μL of 20 ng of template DNA, 5 μL of Multiplex Master Kit (Qiagen) for mixed primers, and Top Taq Master Kit (Qiagen) for Gf01 and 1.5 μL of water nuclease free (Qiagen). We followed the PCR thermal profiles and mixes described in Rodríguez‐Bardía et al. (2022). The PCR was performed in a Veriti thermocycler (Applied Biosystems, Foster City, CA, USA). Capillary electrophoresis was performed on a 3500 genetic analyzer (Applied Biosystems) using Hi‐Di formamide and GeneScan 500 LIZ dye size standard (Applied Biosystems). Multilocus genotypes were scored using GeneMarker 1.91 (SoftGenetics, State College, PA, USA).

### 2.3 Acoustic Analyses

We analyzed all songs using Raven Pro 1.6 software (Cornell Lab of Ornithology, Ithaca, NY, USA). Since this species can produce up to 33 different song types in its repertoire (Sandoval et al. 2014, 2016), we classified each song according to its structure in different types. Solo songs consist of three sections; the first includes a series of high‐frequency elements, followed by a middle section of frequency‐modulated elements, and a final short trill (Sandoval et al. 2016). The first and final sections could present subtle variations because different individuals add or omit elements, but both sections maintain similar fine structural details (Sandoval et al. 2014). Thus, we define different song types based on the variety and form of the middle elements following the classification of the sound library for white‐eared ground‐sparrow available in LEUCA and used in previous studies (Sandoval et al. 2014, 2015, 2016; Méndez et al. 2020). From the songs, we measured the following acoustic characteristics: duration (s), minimum frequency (kHz), maximum frequency (kHz), peak frequency (frequency of maximum amplitude) (kHz), and the number of elements (an element or note is the simplest individual sound that birds produce; Brenowitz et al. 1997). We used different windows to obtain the measurements: the spectrogram window was used to identify and classify sounds, the power spectrum was used to measure frequency characteristics with a threshold of −30 dB relative to the vocalization peak, and the oscillogram was used to measure the temporal characteristics of sounds. Spectrograms were constructed using a Hann window with a size of 512 samples, a 3 dB bandwidth of 124 Hz, a time grid with an overlap of 50% and a hop size of 5.80 ms, and a frequency grid spacing with a DFT size of 512 samples and a grid spacing of 86.1 Hz.

### 2.4 Morphometric Measurements

We measured six morphological traits: tarsus length (from the intertarsal joint to the middle of the foot sole), tail length, wing chord length (unflattened), exposed culmen length (from the tip of the beak to the base of the skull), beak width (at the beak gape), and beak depth (measured at right angles to the point on the lower mandible where the feathers end) following the protocol described in Sandoval and Mennill (2013). We used a dial caliper (model: SPI Plastic Caliper 150 mm, AVINET, NY, USA) to get the bill and tarsus measurements and a metal wing ruler (model: WING15ECON, AVINET, NY, USA) to measure wing chord and tail length.

### 2.5 Statistical Analyses

#### 2.5.1 Genetic Analyses

Mme7 is a sex‐linked marker, and thus females only have one allele (hemizygotes); therefore, we coded it as a missing allele for females and juveniles that cannot be accurately sexed in further analyses as suggested in previous studies (Rasner et al. 2004; Rodríguez‐Bardía et al. 2022) to avoid including extra alleles that may bias allele frequency estimates. We genotyped 76 individuals: 28 males from period 1, 25 males from period 2, 14 females from period 1, and 9 females from period 2. We estimated deviation for the Hardy–Weinberg equilibrium as well as for linkage disequilibrium using Arlequin 3.5.2. We calculated expected heterozygosity ($H_{e}$), observed heterozygosity ($H_{o}$), allelic richness ($A_{r}$), and inbreeding coefficients ($F_{\text{IS}}$) for each population and time period. We estimated confidence intervals for *F*_*IS*_ values using 9999 bootstraps as implemented in the “hierfstat” package in R (R Core Team 2020). To compare differences in heterozygosity for the same populations between time periods, we used *z*‐scores from a Wilcoxon signed rank test as implemented in the “coin” package in R (R Core Team 2020). To estimate changes in genetic structure among populations over a decade, we calculated Nei's $G_{\mathrm{st}}$ coefficient among populations within time periods, using 9999 bootstrap values to estimate 95% confidence intervals using the “mmod” package in R. We assumed that nonoverlapping confidence intervals indicated significant differences in genetic structure between periods. We used an analysis of molecular variance (AMOVA) as implemented in the “poppr” package in R to partition genetic variance among and within time periods. A hierarchical population structure was assumed, with populations nested within time periods. We used 9999 permutations to assess the significance of variance components. Using the Bayesian clustering algorithm in Structure 2.3.4, we assessed the potential genetic clustering of individuals into clusters (K) based on their allele frequencies (Pritchard et al. 2000). To determine the most likely number of K clusters, we used the same parameters as previously used in a study with data from the same populations (Rodríguez‐Bardía et al. 2022). We used correlated allele frequencies and an admixture model with 300,000 Markov chains and a burning of 30,000 chains. We grouped individuals in 1–6 clusters with 20 repetitions for each cluster (Rodríguez‐Bardía et al. 2022). To determine the most likely number of K clusters, we used Structure Harvester 0.6.94 (Evanno et al. 2005). After determining the most likely number of K clusters, we used this K value to conduct another STRUCTURE analysis with 1,000,000 Markov chains and a burn‐in of 100,000 chains to ensure proper chain mixture (Rodríguez‐Bardía et al. 2022).

#### 2.5.2 Acoustic Analyses

To analyze the effect of time on acoustic measurements, we analyzed 10,707 solo songs. We conducted a principal component analysis (PCA) using the “FactoMineR” R package (Lê et al. 2008) to condense the five song measurements into three principal components with eigenvalues > 1, which explained 76% of the total variance. The first principal component (PC1) correlated with duration and number of elements, while the second (PC2) correlated with minimum and maximum frequency, and the third (PC3) correlated with the frequency of maximum amplitude (Table 1). We performed linear mixed models (GLMM) using the “lme4” package (Bates et al. 2015) to determine the effect of populations and time periods on the variation of acoustic traits. Specifically, we included in the model two independent variables: populations (four levels: MTV, HDA, UCR, and JBL), time periods (two levels: period 1 and period 2), and the interactions between both variables. Each of the three principal components (PC1, PC2, and PC3) of the song was included as a response variable in the model, and the territory inside each population was a random factor. We additionally carry out Bonferroni *post hoc* tests when the model shows significant differences in pairwise comparisons between the main effects and the two‐factor interactions.

**TABLE 1:** Results of principal component analysis of solo songs of the white‐eared ground‐sparrow *Melozone leucotis* .

Song measurements	Principal component		
	PC1	PC2	PC3
Duration (s)	0.82*	0.25	−0.09
Minimum frequency (kHz)	−0.26	0.78*	0.24
Maximum frequency (kHz)	0.55	−0.58*	0.30
Frequency of maximum amplitude (kHz)	0.11	0.09	0.93*
Number of elements (*n*)	0.71*	0.43	−0.18

*Note:* The asterisk indicates the variables that have a larger contribution in each component.

#### 2.5.3 Morphological Analyses

To analyze the effect of time on body measurements, we measured 87 individuals, 55 males and 32 females. We conducted a principal component analysis (PCA) using “FactoMineR” to condense the six body measurements into two variables with eigenvalues > 1, which together explained 60% of the total variance. The first principal component (PC1) correlated with tarsus length, tail length, wing chord length, and beak depth, and the second principal component (PC2) correlated with exposed culmen length and beak width (Table 2). We performed a generalized linear mixed model (GLMMs) with a gamma error distribution, using “lme4”. In these analyses we included populations (MTV, HDA, UCR, and JBL), time periods (period 1 and period 2), and the interactions between both variables as independent variables; each of the two principal components (PC1 and PC2) as the response variable, and the territory inside each population in which individuals were captured as a random factor. We also performed models for females and males because males are larger than females (Sandoval and Mennill 2013). We carried out Bonferroni post hoc tests when the model showed significant differences in pairwise comparisons between the main effects and the two‐factor interactions.

**TABLE 2:** Results of principal component analysis of body measurements of white‐eared ground‐sparrow *Melozone leucotis* .

Body measurements	Principal component	
	PC1	PC2
Tarsus length (mm)	0.73*	0.28
Tail length (mm)	0.77*	0.13
Wing chord length (mm)	0.75*	−0.15
Exposed culmen length (mm)	0.02	0.83*
Beak width (mm)	0.38	−0.75*
Beak depth (mm)	0.65*	0.11

*Note:* The asterisk indicates the variables that have a larger contribution in each component.

Finally, to determine if the divergence between different traits (i.e., genetic, acoustic, and morphology) was related, we conducted a partial Mantel test to evaluate the strength of the correlation between variables using 10,000 permutations as implemented in the “vegan” package in R (Oksanen et al. 2012). Females in this analysis were not considered because they do not sing (Sandoval and Mennill 2014; Sandoval et al. 2016). We used 46 males with data on the three variables. First, we performed a PCA to condense the six body measurements and another PCA to condense the five song measurements of the males. Using the PCAs, we then obtained acoustic and morphological distance matrices created from Euclidean distances of mean population attributes across time periods and the genetic distance matrix from Gst values.

## 3 Results

### 3.1 Genetic Diversity

We found no significant evidence of linkage disequilibrium in any of the loci. Two loci deviated significantly from HWE (Gf01 and Gf05), but excluding these loci from our analysis yielded consistent results; thus, we decided to retain the loci to improve our statistical power. At UCR, we found a greater observed heterozygosity ($H_{o}$) in period 1 compared to period 2 (*z* = 1.95, *p* = 0.05), whereas the genetic diversity of the other populations did not differ between time periods (all comparisons: *z* = 1.01, *p* > 0.05) (Table 3). In HDA and JBL populations, the expected heterozygosity ($H_{e}$) was lower in period 1 compared to period 2 (*z* = −2.37, *p* < 0.05; same values for both populations), whereas the other populations did not differ in this parameter (all comparisons: *z* = 0.68, *p* > 0.05) (Table 3). In period 2, HDA, JBL, and UCR showed significant inbreeding coefficients (Table 3).

**TABLE 3:** Genetic diversity estimates for four populations studied in two time periods (2011–2012 and 2018–2022) for white‐eared ground‐sparrow *Melozone leucotis* in Costa Rica.

Population	Time period	*n*	*H*_*o*_		*H*_*e*_		*F*_*is*_		*A*_*r*_
			Mean	SD	Mean	SD	Mean	SD	
MTV	2011–2012	12 (4)	0.64	0.09	0.60	0.09	−0.09	0.05	3.16
MTV	2018–2022	7 (2)	0.54	0.11	0.58	0.11	0.09	0.07	3.09
HDA	2011–2012	7 (3)	0.59	0.12	0.49*	0.10	−0.20*	0.05	2.72
HDA	2018–2022	11 (3)	0.59	0.10	0.71*	0.05	0.18*	0.12	3.59
JBL	2011–2012	11 (5)	0.55	0.08	0.52*	0.08	−0.07*	0.09	2.64
JBL	2018–2022	12 (4)	0.57	0.11	0.68*	0.06	0.22*	0.12	3.48
UCR	2011–2012	12 (2)	0.617*	0.13	0.50	0.11	−0.26*	0.05	2.76
UCR	2018–2022	4	0.321*	0.12	0.59	0.07	0.48*	0.20	3.14

*Note:* Mean ± SD of observed heterozygosity ($H_{o}$), expected heterozygosity ($H_{e}$), inbreeding coefficient ($F_{\text{is}}$), and allelic richness ($A_{r}$). In parentheses, the number of females in each population. The asterisk (*) indicates a statistical difference between time periods, based on *z*‐scores from a Wilcoxon range test.

Abbreviations: HDA, Getsemaní Heredia; JBL, Jardín Botánico Lankester; MTV, Estación Biológica Monteverde; UCR, Universidad de Costa Rica.

**FIGURE 2:**
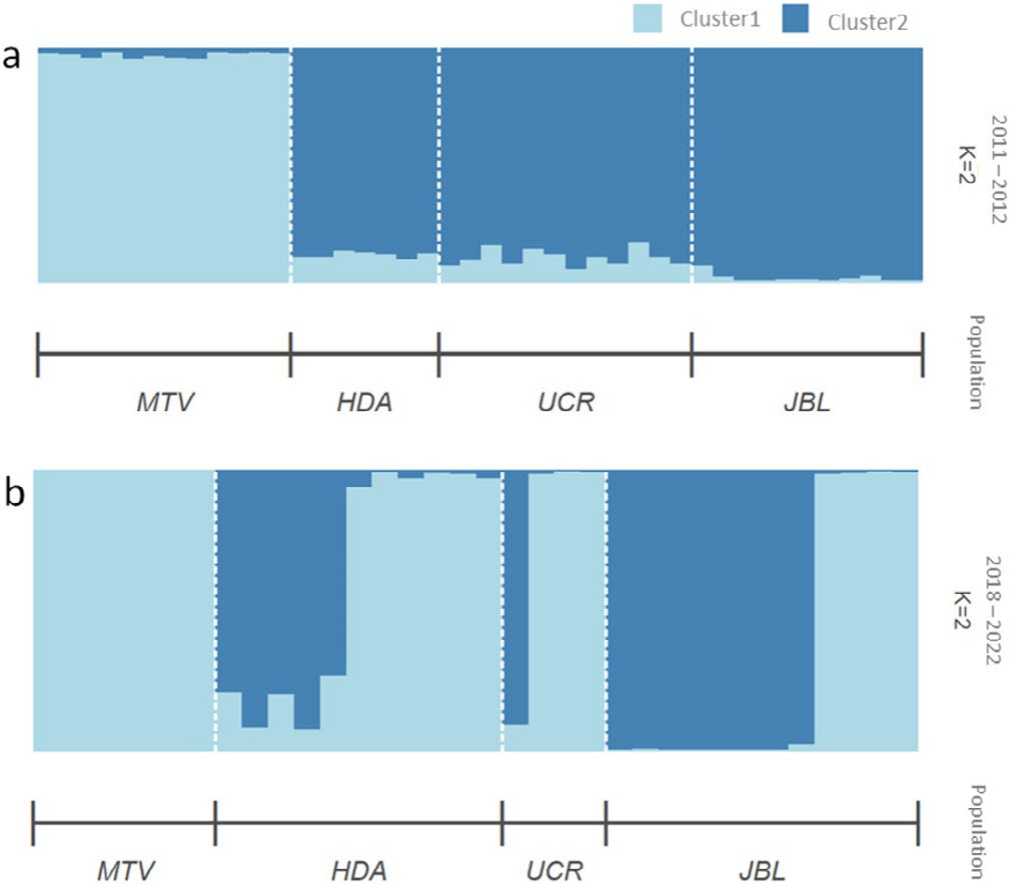
STRUCTURE admixture plots for (a) 2011–2012 populations of white‐eared ground‐sparrow *Melozone leucotis* grouped into K = 2 clusters, and (b) 2018–2022 populations of white‐eared ground‐sparrow *Melozone leucotis* grouped into K = 2 clusters. Plots are sorted by sampling location; each bar represents one individual, and each color represents a cluster. HDA, Heredia; JBL, Jardín Botánico Lankester; MTV, Monteverde; UCR, Universidad de Costa Rica.

Genetic diversity was significantly structured in the metapopulation; however, structure did not differ between time periods: period 1 ($G_{\mathrm{st}}$ = 0.07, 95% CI = 0.06 and 0.09) and period 2 ($G_{\mathrm{st}}$ = 0.07, 95% CI = 0.06 and 0.09). AMOVA results showed that 12% of total genetic variation was partitioned among individuals in different time periods. There were significant differences in allele frequencies between periods (i.e., period 1 vs. period 2; $\phi_{\mathrm{ct}}$ = 0.120, *p* < 0.05), as well as among populations within time periods ($\phi_{\mathrm{sc}}$ = 0.15, *p* < 0.001) (Table S1). In both time periods, the Evanno method suggested that populations were likely grouped into two clusters (i.e., K = 2) (Figure S1). In period 1, all MTV individuals were assigned to cluster 1, while nearly all HDA, UCR, and JBL individuals were assigned to cluster 2 (Figure 2). In period 2, individuals were also grouped into K = 2 clusters. Cluster 1 contained half of the HDA, UCR, and JBL populations, while cluster 2 contained the other half (Figure 2).

### 3.2 Acoustic Characteristics of Songs

**FIGURE 3:**
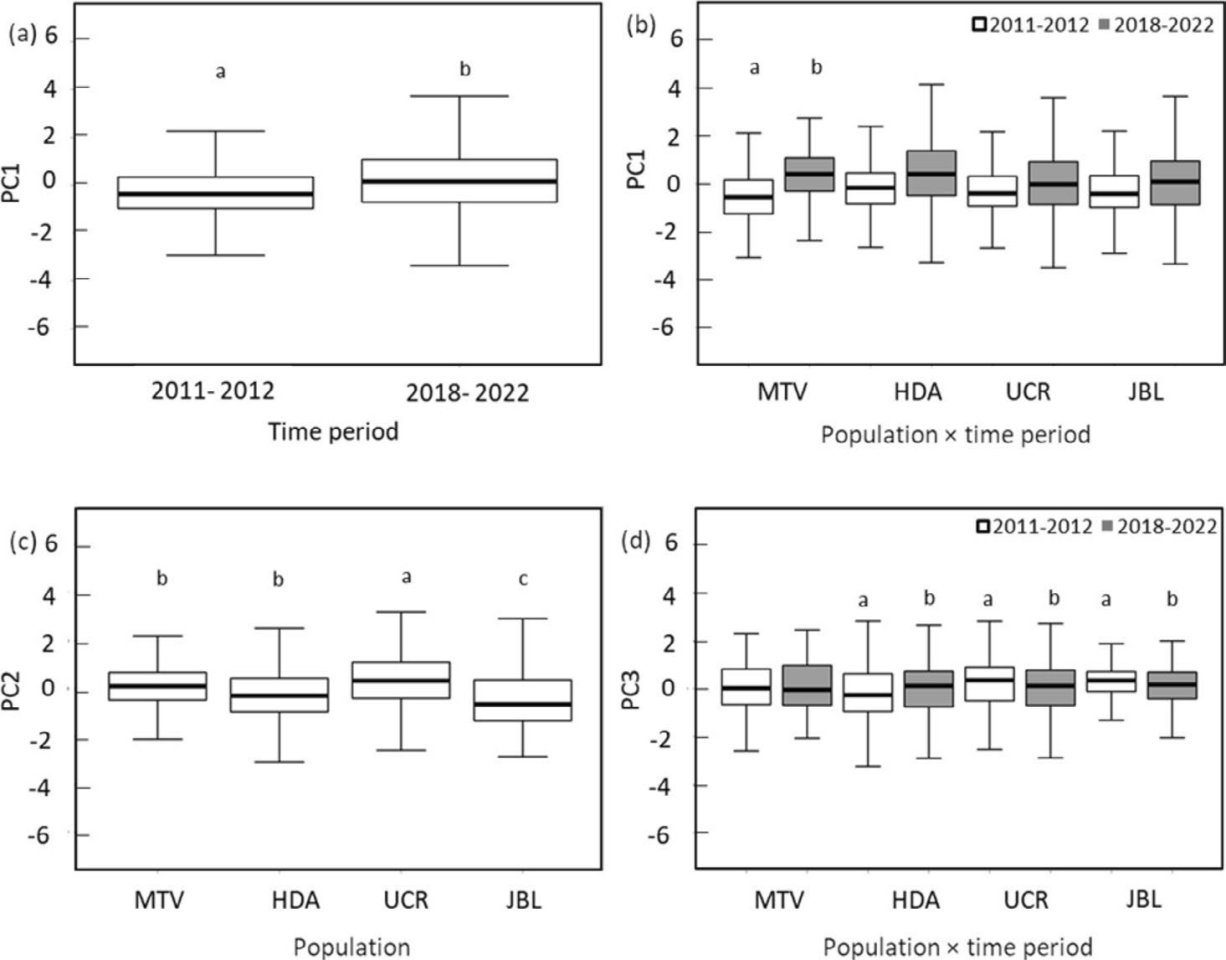
Box plots of the variation in acoustic characteristics of white‐eared ground‐sparrow *Melozone leucotis* , based on linear mixed‐effects models. The responses are measured as principal component scores. Comparison of song duration and number of elements between time periods (a) and across populations and time periods (b), based on PC1 values; comparison of the minimum and maximum frequency, which correlated with PC2 (c); and comparison of the frequency of maximum amplitude, which correlated with PC3 (d). The box plot shows the median (central horizontal line), the 75th and 25th percentiles (top and bottom of the box), and the maximum and minimum values (top and bottom whiskers). Different letters indicate significant differences between time periods or populations between time periods. Lack of letters indicates that no significant differences were detected in the post hoc tests.

We found that the duration and number of elements (PC1) were affected by the time period and by the interaction between the population and the time period (Table 4). In MTV, songs increased in duration and number of elements in ‘period 2’ (Figure 3). The minimum and maximum frequency (PC2) were not affected by time, but the other acoustic variables differed significantly among populations (Table 4). UCR had a higher minimum frequency and lower maximum frequency than MTV and HDA, while JBL had a lower minimum frequency and higher maximum frequency compared to other populations (Figure 3). The frequency of maximum amplitude (PC3) showed significant differences in the interaction population × time period (Table 4). The songs increased the frequency of maximum amplitude in HDA period 2, relative to period 1, whereas UCR and JBL decreased in period 2 compared to period 1.

**TABLE 4:** Comparison of acoustic characteristics of solo songs between two time periods (2011–2012, 2018–2022) in four populations and their interaction with white‐eared ground‐sparrows *Melozone leucotis* , using linear mixed‐effect models.

	PC1			PC2			PC3		
	df	*F*	*p*	df	*F*	*p*	df	*F*	*p*
Population	3	1.67	0.18	3	10.58	< 0.001	3	1.90	0.13
Time period	1	8.84	< 0.05	1	2.86	0.09	1	1.38	0.24
Population × time period	3	2.73	< 0.05	3	1.04	0.38	3	3.65	< 0.05

*Note:* PC1 correlates with the duration and number of elements, PC2 correlates with the minimum and maximum frequency, and PC3 correlates with the frequency of maximum amplitude.

### 3.3 Morphology

**FIGURE 4:**
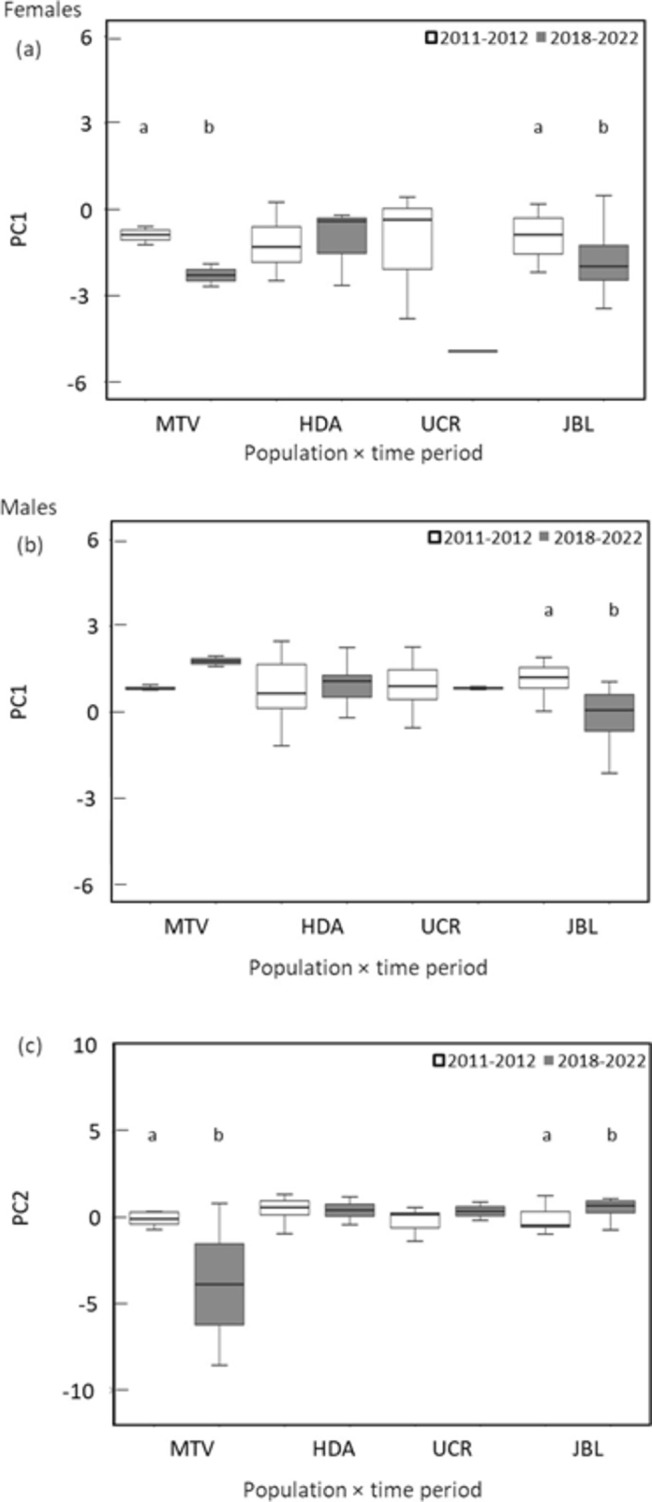
Box plots of the variation in morphology of white‐eared ground‐sparrow *Melozone leucotis* , based on generalized linear mixed models. The responses are measured as principal components scores; Comparison of tarsus length, tail length, wing chord length, and beak depth, which correlated with PC1 of females (a), comparison of tarsus length, tail length, wing chord length, and beak depth, which correlated with PC1 of males (b); and comparison of the exposed culmen length and beak width, which correlated with PC2 of males (c). The box plot shows the median (central horizontal line), the 75th and 25th percentile (top and bottom of the box), and the maximum and minimum values (top and bottom whiskers). Different letters indicate significant differences between time periods or populations between time periods. Lack of letters indicates that no significant differences were found in the post hoc tests.

We found a significant interaction between population and time period in females for tarsus length, tail length, wing chord length, and beak depth (PC1) (Table 5). These morphological variables represented by the PC1 decreased between periods 1 and 2 for MTV and JBL (Figure 4). The exposed culmen length and beak width (PC2) did not show significant differences (Table 5) among populations or time periods. For males, we also found a significant interaction between population and time period for PC1 (Table 5). Tarsus length, tail length, wing chord length, and beak depth were smaller in JBL period 2 compared to period 1 (Figure 4). The exposed culmen length and beak width (PC2) were affected by the interaction between population and time period (Table 5). Birds in MTV period 2 had shorter exposed culmen and bigger beak width than in period 1, and birds in JBL period 2 had bigger exposed culmen length and smaller beak width than in period 1 (Figure 4).

**TABLE 5:** Comparison of body measurements in two periods of time (2011–2012 vs. 2018–2022) in four populations and their interaction of white‐eared ground‐sparrows *Melozone leucotis* , based on generalized linear mixed models.

	Females						Males					
	PC1			PC2			PC1			PC2		
	*X* ^2^	df	*p*	*X* ^2^	df	*p*	*X* ^2^	df	*p*	*X* ^2^	df	*p*
Population	0.75	3	0.86	1.72	3	0.63	2.28	3	0.52	0.62	3	0.89
Time period	0.24	1	0.63	1.25	1	0.26	0.001	1	0.97	0.005	1	0.94
Population × time period	31.73	3	< 0.001	2.68	3	0.44	27.14	3	< 0.001	10.15	3	< 0.05

*Note:* PC1 correlates with tarsus length, tail length, wing chord length, and beak depth; PC2 correlates with exposed culmen length and beak width.

### 3.4 Relationship Between Variables

**FIGURE 5:**
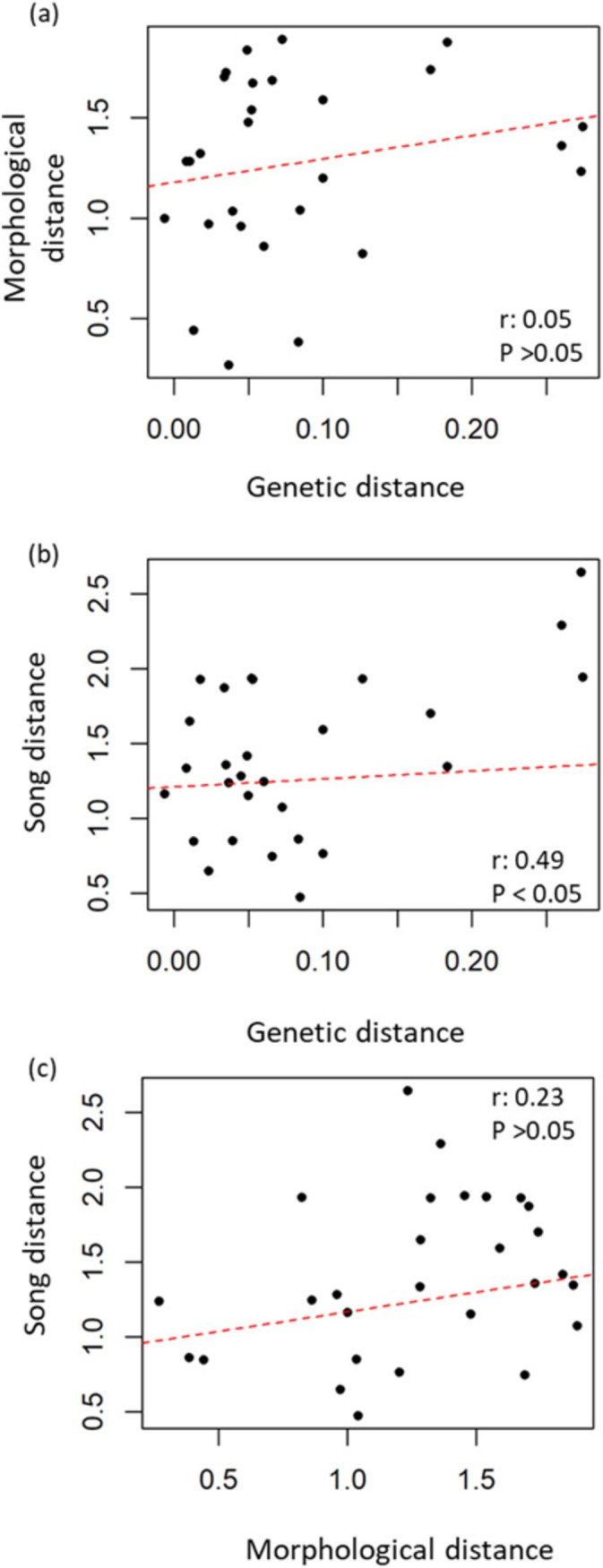
Correlations between different sets of variables in white‐eared ground‐sparrow *Melozone leucotis*. (a) Song and morphology distances, (b) morphology and genetic distances, and (c) song and genetic distances. Regression lines are shown, but statistical significance was evaluated using Mantel tests.

When acoustic differences were controlled, the partial Mantel test revealed a non‐significant association between genetic and morphological distances (*r* = −0.06, *p* = 0.40; Figure 5). Similarly, we found no significant correlation between morphology and acoustic distances while accounting for genetic differences (*r* = 0.24, *p* = 0.10; Figure 5). However, we found a significant correlation between genetic distances and acoustic distances when morphological distances were accounted for (*r* = 0.49, *p* < 0.05; Figure 5).

## 4 Discussion

Over the 10‐year period between samplings, urbanization around our sampled populations increased (Biamonte et al. 2011; Rodríguez‐Bardía et al. 2022). Large areas of natural or semi‐natural habitats were eliminated, leading to greater noise, increased population isolation, and a likely decline in habitat quality (Joyce 2006; Biamonte et al. 2011; Méndez et al. 2020). These changes in the urbanscape align with our findings of increased inbreeding and changes in acoustic and morphological traits over the same period. However, only genetic and song distances were correlated. These results are likely explained by the interaction between declining habitat quality and barriers to gene flow caused by rapid urban development in the sampling area (Biamonte et al. 2011; Juárez et al. 2020, 2022; Rodríguez‐Bardía et al. 2022), particularly given the low mobility, isolation, and specific habitat requirements of the white‐eared ground‐sparrow (Juárez et al. 2020; Rodríguez‐Bardía et al. 2022).

### 4.1 Genetics

We found an increase in inbreeding in three populations exposed to rapid urbanization in the Costa Rican Central Valley (HDA, JBL, and UCR). Inbreeding is predominantly caused by consanguineous matings, and its extent is proportional to a reduction in effective population sizes or a reduction in the dispersal of individuals among populations (Frankham et al. 2002). Therefore, the increase in inbreeding could be explained by urban expansion, which rapidly transforms natural ecosystems into homogenized environments that limit resources to small suboptimal patches of vegetation (Biamonte et al. 2011; Fahrig and Rytwinski 2009; Miles et al. 2019). These drastic changes brought about by urbanization restrict the movement of birds between populations (Rodríguez‐Bardía et al. 2022). Consequently, small and isolated populations increase the likelihood of mating with relatives (Wright et al. 2008). A similar increase in inbreeding associated with isolation was reported for the Taita thrush, *Turdus helleri* (Lens et al. 2000), and the striped tit‐babblers, *Mixornis gularis* (Tan et al. 2018).

Results from the AMOVA analysis revealed that population structure has a greater influence on spatial genetic structure than time periods. This result is consistent with the isolation by distance and the urban resistance to gene flow previously reported for this species (Rodríguez‐Bardía et al. 2022). Limited gene flow is expected for species with low mobility and specific habitat requirements, such as the white‐eared ground‐sparrow (Delaney et al. 2010; Rodríguez‐Bardía et al. 2022; Soulé et al. 1988). However, STRUCTURE clustering suggests that some movement of individuals may still occur between populations; alternatively, allele frequencies are becoming homogenized through drift, resulting in a few individuals being clustered together by STRUCTURE even though they are in separate isolated populations.

The indirect evidence of limited gene flow found in our study could also be a result of the interaction between philopatry, territoriality, and the lack of suitable habitat for populations of ground sparrows in an urban setting. Philopatry and territoriality maintain individuals within a population, thereby decreasing gene flow and increasing genetic differentiation between populations (Bounas et al. 2018; Rodríguez‐Bardía et al. 2022). In urbanized sites, ground‐sparrow males increase their territories to likely search for additional food sources (Juárez et al. 2020). This foraging behavior may prompt some males to migrate when resources are scarce, explaining the movement of individuals detected in our Bayesian clustering.

On the contrary, individuals inhabiting isolated populations often develop local adaptations, such as different dialects and variations in the frequency and duration of songs, as shown for the white‐eared ground‐sparrow (Sandoval et al. 2014, 2015, 2016; Juárez et al. 2020; Méndez et al. 2020). These adaptations could further reduce individual dispersal between populations, thus contributing to increased genetic differences among remaining populations (Morhina et al. 2017). For example, in white‐crowned sparrows ( *Zonotrichia leucophrys* ), spatial genetic structure was correlated with song dialects because song dialects act as barriers for migrant males, increasing differences in allele frequencies among populations (MacDougall‐Shackleton and MacDougall‐Shackleton 2001).

We only observed a significant decline in genetic diversity between time periods in the most urbanized location ($H_{o}$ UCR period 1: 0.62 vs. $H_{o}$ UCR period 2: 0.32). This result is consistent with previous research indicating that urbanization reduces genetic diversity in wren‐tits *Chamaea fasciata* , side‐blotched lizards *Uta stansburiana* , western skinks *Plestiodon skiltonianus*, and western fence lizards *Sceloporus occidentalis* inhabiting more isolated habitat patches (Delaney et al. 2010). Consequently, cities and human‐made structures appear to act as barriers to gene flow (Rodríguez‐Bardía et al. 2022), limiting the movement of individuals among vegetation remnants within cities. Therefore, in the span of a decade, we were able to document a decrease in genetic diversity of this bird species in the most urbanized population. The presence of dialects and assortative mating within populations may also restrict the entry of new males, further limiting gene flow between populations and consequently reducing population genetic diversity (Newton 1992; Camacho‐Alpízar et al. 2018). Our findings should be interpreted with caution due to the small number of individuals collected at UCR during period 2. Although our sample was fully random, discrepancies in population size could bias our inbreeding estimates. For example, we could have randomly captured individuals that were closely related, which would result in an increase in our estimates of the inbreeding coefficient.

### 4.2 Acoustics

Differences in solo songs of white‐eared ground‐sparrows are concordant with previous studies that reported acoustic differences as a possible adaptation to urban noise (Méndez et al. 2020; Sandoval et al. 2016; Sandoval and Mennill 2014; Bonilla‐Badilla 2021). However, we additionally discovered a correlation between acoustic and genetic distances while controlling for morphology. These results reaffirmed that urban development could become an important barrier among populations that isolate and limit the movements of individuals (Rodríguez‐Bardía et al. 2022; Sosa‐López et al. 2013). The same relationship between genetic and acoustic distances has been reported in populations of rufous‐naped wrens ( *Campylorhynchus rufinucha* ), which lost connectivity by historical isolation due to a marine barrier during the formation of the Isthmus of Tehuantepec in the late Pleistocene (Vázquez‐Miranda et al. 2009). The correlation between genetic and acoustic distances and the pattern of isolation by distance previously reported in the white‐eared ground‐sparrow (Rodríguez‐Bardía et al. 2022) suggest an important role of cultural drift or sexual selection (Camacho‐Alpízar et al. 2018; Irwin et al. 2008; West‐Eberhard 1983). White‐eared ground‐sparrows use solo songs for female attraction, and males, after learning songs, are not able to learn new songs (Sandoval et al. 2016; Bonilla‐Badilla 2021). Thus, if females choose specific acoustic traits, song types, or young males imitate specific songs in each population, a single phenotype will be selected across generations (West‐Eberhard 1983). For example, female great tits, *Parus major* , prefer males that emit song types with higher frequencies in noisy environments (Halfwerk et al. 2011). In this context, the higher frequencies in urbanized populations and the different dialects among populations of white‐eared ground‐sparrows (Méndez et al. 2020; Sandoval et al. 2016; Sandoval and Mennill 2014; Bonilla‐Badilla 2021) may show the effect of sexual selection acting differently in each population.

### 4.3 Morphology

The female body size of white‐eared ground sparrows decreases in MTV and the JBL as well as in males of JBL population between sampling periods. Body size often decreases in response to a decrease in food availability and quality (Goodman et al. 2012; Salewski et al. 2014; Yom‐Tov and Geffen 2011). Introduced species such as rats and mice may compete for food resources with white‐eared ground sparrows. A similar reduction in body size was observed in the lesser sheathbills, *Chionis minor* , in Marion Island, due to the introduction of rats and cats that eat terrestrial macro‐invertebrates, the sheathbills primary food resource (Huyser et al. 2000). In white‐eared ground‐sparrows reduction in body size may also be caused by lower‐quality food as a result of urbanization (Mennechez and Clergeau 2006). Higher predation risks could also play an important role in size reduction because smaller individuals may be better able to avoid cats and other predators (Seress et al. 2011). Given that sparrows are a common prey of cats (Seress et al. 2011; Yom‐Tov and Geffen 2011), this would be an important adaptation (or selection pressure) for ground sparrows in highly urbanized sites.

In JBL males, the exposed culmen length increased and beak width decreased between sampling periods. Due to the widespread use of bird feeders, the trend toward longer beaks has been linked to the consumption of larger prey or generalized feeding (Bosse et al. 2017; Hüppi and Geiger 2022; Rolshausen et al. 2009). In great tits, *Parus major* , longer beaks correlated with fitness because birds with longer beaks are able to feed more efficiently from bird feeders (Bosse et al. 2017). The width is another characteristic of the beak that is affected by the availability of some food types (Badyaev et al. 2008). For example, the house finches *Haemorhous mexicanus* that inhabit urban populations in Arizona have a narrower beak, and thus less bite force, because they consume softer human food (Badyaev et al. 2008; de León et al. 2011; Hüppi and Geiger 2022). In contrast, in MTV, males decrease their exposed culmen length and increase their beak width. The increase in beak width suggests that birds are likely consuming larger and tougher food, which would require an increase in bite force (de León et al. 2011; Hüppi and Geiger 2022). Differences in beak lengths between populations may also be the result of individuals consuming novel foods, regardless of the morphology of the species, indicating adaptation toward exploiting different food resources (de León et al. 2011; Grant and Grant 2002).

Comparing four populations of white‐eared ground‐sparrows over a decade revealed changes in levels of inbreeding, spectro‐temporal song characteristics, and morphological traits.

Interestingly, we found a positive relationship between genetic divergence among populations and song divergence. This correlation suggests that urbanization may act as a barrier for white‐eared ground sparrows. Alternatively, it may be explained by cultural drift or sexual selection, as changes in acoustic signals associated with genetic divergence can occur if selection for song preferences persists within populations (Irwin et al. 2008; Sathyan et al. 2017). The relationship between genetic and phenotypic traits is often slow and complex due to the simultaneous action of various forces (Irwin et al. 2008; Carnicer et al. 2009; Sathyan et al. 2017). Only long‐term studies in urban environments shed light on the processes of adaptation of different species to the drastic changes imposed by urbanization, since species with different life history traits may respond and adapt in different ways to the same environmental changes (Rodríguez‐Bardía et al. 2022).

## Author Contributions

**Luis Cueva:** conceptualization (equal), data curation (equal), formal analysis (equal), investigation (equal), methodology (equal), writing – original draft (equal), writing – review and editing (equal). **Eric J. Fuchs:** data curation (equal), formal analysis (equal), investigation (supporting), methodology (supporting), supervision (equal), writing – review and editing (equal). **Gilbert Barrantes:** conceptualization (equal), writing – review and editing (equal). **Ruth Madrigal‐Brenes:** formal analysis (supporting), investigation (supporting), supervision (supporting). **Luis Sandoval:** conceptualization (equal), data curation (equal), formal analysis (equal), funding acquisition (equal), investigation (equal), supervision (equal), writing – review and editing (equal).

## Conflicts of Interest

The authors declare no conflicts of interest.

## Supporting information


Appendix S1.

## Data Availability

Data are available at https://doi.org/10.5281/zenodo.10472250.
