# Supplementary material for: Effect of Spatial and Temporal Urban Isolation on the Genetic Diversity, Acoustic Variation, and Morphological Characteristics of an Urban Survivor Bird Species
Source: Ecol Evol. 2025 Apr 28;15(4):e70972. doi: 10.1002/ece3.70972 (PMC12037210; doi:10.1002/ece3.70972)
Supplement: Supplementary file 1 — Appendix S1. [file ECE3-15-e70972-s001.docx]

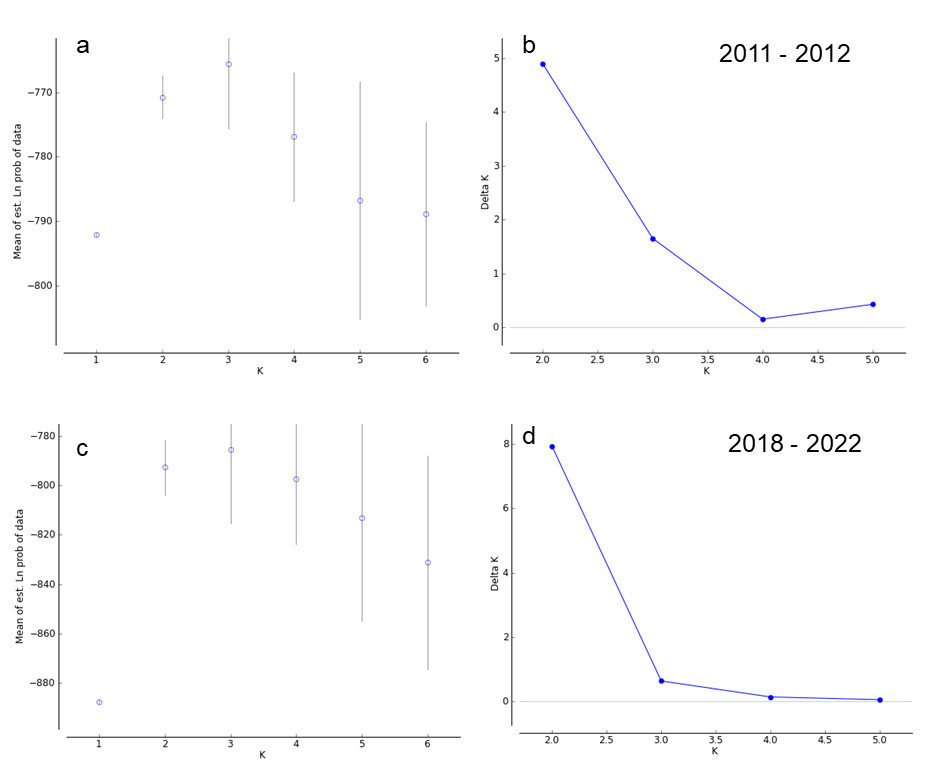


Supplementary Figure S1. Structure Harvester results: mean L(K) (a and c) and Delta K (b and d) for White-eared ground-sparrow in both periods 2011-2012 (a and b) and 2018-2022(c and d).

Supplementary Table S1. Analysis of molecular variance (AMOVA) FOR SSR markers for populations of White-eared ground-sparrow in Costa Rica.

| Source of variation | df | Sum of Squares | Mean of Squares | Percent of variation | Fixation indices |
| --- | --- | --- | --- | --- | --- |
| Between time periods | 1 | 15.413 | 15.413 | 12.09 | $F_{ct}$ = 0.12*** |
| Among populations within time periods | 6 | 30.616 | 5.102 | 13.54 | $F_{sc}$ = 0.154*** |
| Within populations | 43 | 103.411 | 2.404 | 74.361 | $F_{st}$ = 0.256* |
| Total | 50 | 149.441 | 2.988 |  |  |
| ***: p<0.001, *: p<0.05 | | | | | |
